# Strategic Management of Bleeding Small Bowel Gastrointestinal Angiodysplasias (GIADs): A 12 Year Retrospective Review in a Veteran Population and Cost Comparison

**DOI:** 10.3390/diagnostics13030525

**Published:** 2023-01-31

**Authors:** Mario Gutierrez, Chandrasekhar Kesavan, Anjali Das, Christian S. Jackson, Richard M. Strong

**Affiliations:** 1 Department of Gastroenterology, VA Loma Linda Veterans Healthcare System, Loma Linda, CA 92354, USA; 2 Department of Medicine, Loma Linda University, Loma Linda, CA 92357, USA; 3 Musculoskeletal Disease Center, VA Loma Linda Veterans Healthcare System, Loma Linda, CA 92354, USA; 4 School of Medicine, University of California, Riverside, CA 92521, USA

**Keywords:** capsule endoscopy, SST, iron, GIADs

## Abstract

Background: Gastrointestinal angiodysplasias (GIADs), also known as gastrointestinal angioectasias, are dilated, abnormally thin-walled blood vessels that occur in the mucosa and submucosa throughout the gastrointestinal tract. As a common cause of small bowel bleeding, GIADs have a significant impact on patient’s morbidity and healthcare costs. Presently, somatostatin has been used widely to treat GIADs, but it is unclear if other therapies are as beneficial and cost-effective as somatostatin in managing GIADs. (2) Methods: A retrospective chart review was performed, which included subjects treated with Lanreotide, a somatostatin analog, and other therapies at the VA Loma Linda Healthcare System (VALLHCC) from January 2006 to December 2018. Patients who had symptomatic GIADs were detected by video capsule endoscopy (VCE), a device-assisted enteroscopy (DAE) or, in our case, push enteroscopy (PE) with an Endocuff. (3) Results: Three hundred twelve patients were diagnosed with GIADs. In this group of patients, 72 underwent ablation (endoscopic BICAP) with the addition of Lanreotide (SST), 63 underwent ablation therapy, eight were treated with SST only, 128 received iron replacement only, 25 received iron plus SST therapy, and 61 were observed with no therapy. Each group was followed via their hemoglobin (Hgb) level immediately thereafter, and Hgb levels were then obtained every 3 months for a 12-month period. After ablation therapy, 63 patients maintained stable Hgb levels over the course of the study, suggesting a significant therapeutic effect by controlling active bleeding. The 27 patients receiving ablation +SST therapy did not show improvements when compared to ablation only and the 128 patients who received iron therapy alone. (4) Conclusions: Importantly, 12 years of managing these patients has given us a cost- and time-sensitive strategy to maintain the patients’ Hgb levels and avoid hospital admissions for acute bleeding. Iron treatment alone is effective compared to SST treatment in recovering from GIADs. Eliminating SST treatment from therapeutic intervention would save $89,100–445,550 per patient, depending on the number of doses for private care patients and $14,286–28,772 for VA patients, respectively. A suggested therapy would be to perform DAE on actively bleeding patients, ablate the lesions using a coagulation method, and place the patient on iron. If that fails, gastroenterologists should repeat VCE and perform either PE with Endocuff or balloon enteroscopy (all DAEs).

## 1. Introduction

Gastrointestinal angiodysplasias (GIADs) are the most frequent vascular lesions and the most common cause of small bowel bleeding in older individuals [1,2]. GIADs can be located throughout the digestive tract and are normally found in 1–5% of all patients undergoing endoscopic studies [3,4]. They are most often detected in patients over 60 years of age, with involvement of the small bowel in 57–80% of cases, particularly in the proximal segment (a finding which we have been able to re-confirm [3], using an Endocuff with deep enteroscopy, followed by the colon in 44% and in the stomach in 32% of cases [5,6]. GIADs are found in more than one segment of the GI tract in 60% of cases, and they are responsible for 4–7% of non-variceal upper gastrointestinal bleeding (GIB) [7,8,9].

Clinically, GIADs can manifest in a variety of different ways depending on the number and size of the affected blood vessels, the location of the lesion within the gastrointestinal tract, and the underlying pathophysiology that may be causing the condition. Signs and symptoms also vary depending on if the bleeding is overt or occult (“Gastrointestinal Bleeding.” Mayo Clinic, Mayo Foundation for Medical Education and Research, https://www.mayoclinic.or/diseases-conditions/gastrointestinal-bleeding/symptoms-causes/syc-20372729, accessed on 15 October 2020). For example, GIADs involving the colon can be evidenced by rectal bleeding, ranging from small amounts of blood mixed in stools to bright red blood. One of the most common presentations of GIADs is iron deficiency anemia (IDA), with a prevalence of approximately 61% [10]. Additional symptoms include abdominal pain (frequently after eating), fatigue, diarrhea, constipation, nausea, vomiting, and abdominal distention (bloating).

The risk factors for developing GIADs are age, aortic stenosis, chronic renal diseases [11,12,13,14,15,16,17,18,19], chronic obstructive pulmonary disease, venous thromboembolism, cardiovascular disease, and liver cirrhosis [20,21], although they are most frequently seen in patients over 60 with aortic valve disease and chronic renal disease. An association has also been made between GIADs and coagulopathy disorders such as von Willebrand’s disease [22].

GIADs are dilated communications between veins and capillaries [23]. Histologically, they consist of an accumulation of ectatic, thin-walled veins, venules, and capillaries lined by the endothelium in the mucosa and submucosa [24]. The diagnosis of GIADs is based on the ability to distinguish them from other vascular lesions in the GI tract and visualize them either by device-assisted enteroscopy (DAE) or video capsule endoscopy (VCE).

The pathophysiology of GIADs is not precisely known. Theories for the development of colonic lesions include intestinal smooth muscle contraction causing intermittent obstruction of the sub-mucosal veins of the intestinal wall, causing capillary congestion [1], and mucosal ischemia and chronic hypoxemia [25] as in aortic stenosis [26]. Coagulation disorders have also been suggested as a mechanism. This mechanism could involve changes in von Willebrand factor and tissue-plasminogen activator activity, both leading to increased plasma fibrinolytic activity. However, these mechanisms remain controversial [27]. A case report using Bevacizumab, an anti-vascular endothelial growth factor (VEGF) monoclonal antibody that inhibits tumor angiogenesis, was used in a case report to control bleeding from GIADs (personal communication). No clear etiology has yet been described for the small bowel angioectasias, other than that these lesions seem to predominate in the proximal small bowel (SB).

The incidental finding of non-bleeding GIADs on endoscopy can present a dilemma as to causality [3,28,29]. Occult GI bleeding is the most frequent indication for video capsule endoscopy (VCE), representing 70–75% of cases [30,31], with GIADs demonstrated in 50–60% of such cases [32,33,34], with only 20% being found outside the small bowel in the colon [35]. Small bowel (SB) GIADs are the most common lesion type (57–80%), causing 5% of all GIB cases. In the small bowel, 50–80% of the lesions are in the jejunum and duodenum, with 5–20% in the ileum [36]. The incidence of recurrent bleeding of SB GIADs is greater than that of the colon and stomach [37], with recurrent bleeding in 80% of cases within an average of 10.7 months of follow-up. SB with GIADs in the colon or stomach are four times more likely to have a bleeding recurrence within a year [38], but SB GIADs are the cause of severe visible bleeding in 35% of cases [39].

Visualization of the small bowel is often incomplete with VCE and device DAE, in 10–16% and 40–50%, respectively [40]. These lesions are difficult to diagnose in the small bowel, being smaller than 5 mm, and are often hidden behind mucosal folds of the small intestine and obscured by peristalsis and intestinal detritus. In our institution, the use of Endocuff-assisted push enteroscopy (DAE) has aided in the visualization and treatment of these lesions [3] and is now our method of choice for treatment. 

There are several classifications for GIADs that are now considered using the endoscopic characteristics obtained by VCE or DAE. The most helpful classification for SB GIADs is that of Garcia-Compean [41] (Types 1, 2, 3, and 4). Type 1 lesions are punctuated or patchy with non-pulsatile active bleeding, while Type 2 lesions are non-actively bleeding lesions with stigmata of hemorrhage and are either ulcers, adherent clots, or digested blood debris. Type 3 lesions are bright-red color patchy spots due to intense vascular congestion, often in the absence of other sources of bleeding. Type 3 lesions are thought to be the cause of small bowel bleeding, and they are the most frequent lesions detected at our institution. Type 4 lesions are pale-red color patchy spots that are difficult to discern from artifacts. The most frequent lesion types seen at LLVAHCS are types 1 and 3 lesions. The lacy lesions seen in the colon are not typical of what is seen in the small bowel [3] and are likely pathophysiologically different, in our opinion.

Treatment modalities for bleeding GIADs include argon plasma coagulation (APC), mechanical clip placement, multipolar electrocoagulation (MPEC), laser photoablation, angiography with embolization, surgical resection, and pharmacologic therapy, most of which have been well-described in the literature [24,42]. These treatment modalities are used in three scenarios which depend on patient presentation: hemostatic, prophylactic, and rescue [42]. At our center, the hemostatic modality is ablation with BICAP or APC, the prophylactic modalities are somatostatin (Lanreotide) [42] and iron replacement, and rescue therapies are surgical or radiographic [24]. This study reviews our treatment management strategies for GIADs over a 12 year span (2006 to 2018). It should be noted that diagnosis and treatment with Endocuff were introduced during the last 5 years of our management assessment, which we feel has made a significant impact on our management practices.

Treatment of GIADs places a heavy financial burden on the healthcare industry each year. One study found that the average cost of hospital admission due to small bowel bleeding averaged $40,456 +/− $8773 [43]. The lengths of hospitalizations ranged from 4.3 to 18.2 days, with small bowel bleeding constituting the longest stays within the hospital. After patients leave the hospital, 84.5% of them will require ambulatory visits, and 80% of them will require prescription refills linked to their GI bleeding within 12 months after an inciting upper GI bleeding event [44]. Up to 20% of patients previously hospitalized for GIADs will be readmitted to the hospital within 30 days, most commonly for direct complications related to chronic GI bleeding [45]. Additionally, the rates of hospitalizations due to GIAD complications are increasing exponentially, evidenced by the 309 and 497% increase in hospitalizations for GIADs with and without hemorrhage, respectively, between 2001 and 2011 [46].

## 2. Materials and Methods

### 2.1. Study Design 

A retrospective assessment of veterans presenting with gastrointestinal bleeding found on video capsule endoscopy (VCE) and deep enteroscopy (DE) from January 2006 to December 2018 was reviewed to assess the best strategy for management. Three hundred and twelve symptomatic patients (inpatients and outpatients) were diagnosed with small bowel angiodysplasias (GIADs) via VCE and DE, with or without the Endocuff device. After the initial stabilization of anemias, most of which were non-actively bleeding chronic anemias, and after undergoing upper and lower endoscopies, a video capsule endoscopy (VCE) was obtained. The VCE was frequently done as an outpatient, as most patients were not actively bleeding. The therapies, device-assisted enteroscopy (DAE) or push enteroscopy (PE), ablation, Lanreotide infusions, and iron replacement, were managed in outpatient clinics. Since admissions and re-admissions were difficult to document for just bleeding and not comorbidities, the Charlson Comorbidity Index Score was used to define the health state of each treatment group. Patients were followed prospectively, and data were analyzed retrospectively for treatment effects. 

### 2.2. Treatment and Data Collection 

The treatment efficacy was evaluated by measuring the hemoglobin levels (Hgb) during 12 months of observation after the procedure. Patients were followed in the gastrointestinal clinics. The following data were collected: age, ethnicity, Charlson Comorbidity Index Score (CMDS), and Hgb levels before treatment (initial) and at 3-month intervals. Patient’s treatment groups were separated into the following categories: patients who underwent ablation (BICAP) with or without Lanreotide (SST), ablation only, SST only, Iron (Fe) treatment only, and Fe with SST. Iron therapy was delivered either orally or by intravenous administration when patients would not tolerate oral iron. Lanreotide was given monthly, at a dose from 90–120 mg, for a total of 12 doses. A cost assessment was performed, comparing the cost of VCE DE versus DE with a balloon and comparing the strategies at VALLHCS to see which management strategy was the most cost-effective, DAE with a balloon or Endocuff, with or without Lanreotide.

### 2.3. Statistical Analysis

The data is presented as mean ± SEM. We used the Tukey Post Hoc test to compare differences between the treatment groups. ANOVA analysis was used to evaluate the influence of morbidity in GIADs. SPSS version 21 was used to perform the statistical analysis. A *p* < 0.05 was considered significant between the groups. 

## 3. Results 

The ages of the patients ranged from 63 to 70 years old. The ethnicity of patients was mainly Caucasian and Hispanic. Sixty-three patients had ablation only, 27 patients underwent ablation and received SST, 128 patients had iron therapy only, 25 patients had iron and SST, eight patients had SST only, and 61 patients had no treatment (Table 1). Treatment efficacy was determined by blood Hgb levels. The GIAD patients that were treated with iron therapy showed significant improvement in Hgb levels as early as 1 month because these patients were not significantly bleeding, while the hemoglobin levels in patients treated with ablation plus SST recovered at 6 months. Ablations, iron and SST treatment resulted in a 12-month improvement in Hgb levels. SST treatment alone did not significantly improve the Hgb levels during a 12 month observation period (Table 2). 

The comorbidity in GIADs patients (Charlson Morbidity Index Score, CMDS) indicated that the index was similar between the groups, except for the observed (control with no treatment), which showed low comorbidity. ANOVA analysis was (*p* < 0.05) for interaction between groups with comorbidity as a co-variance (Table 3).

## 4. Discussion

**Figure 1:**
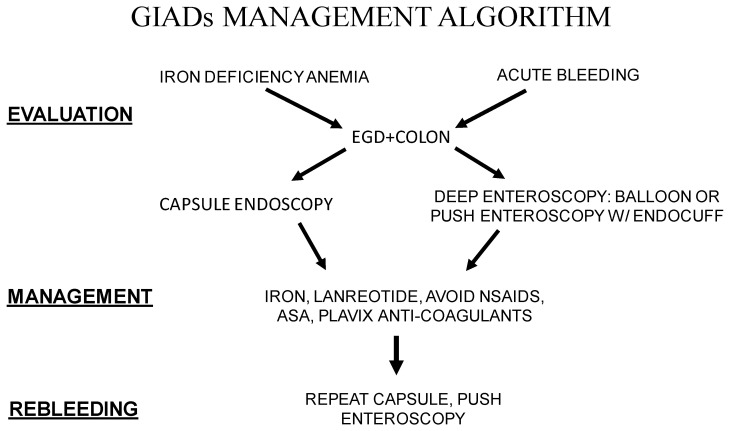
A schematic representation of GIADs management algorithm.

GIADs are one of the most common causes of small-bowel bleeding in patients older than 60 years of age with medical co-morbidities. Patients suffering from GIADs are often fragile and can present acutely or chronically with iron deficiency anemia. Choosing the proper treatment modality depends on the acuteness of presentation and how the co-morbidities affect the ability to aggressively manage the patient (Figure 1). Although it is well established that the diagnosis of these patients requires first screening with upper endoscopy, then colonoscopy, followed by video capsule endoscopy (VCE) when the EGD and colonoscopy are negative. Subsequently, assessment requires a determination of whether direct visualization and treatment are going to be done via DAE, choosing either balloon enteroscopy or push enteroscopy, or in our case, by enhanced enteroscopy with an Endocuff device. 

Actively bleeding lesions are managed with cautery or clipping. Chronic lesions are often managed with iron replacement, with avoidance of barrier breakers and somatostatin analogs. Seeing the lesion that is bleeding is one challenge, but the limitation of treatments imposed by fragility and co-morbidity is also a second challenge. Twelve years of managing these patients has given us a cost- and time-sensitive strategy to maintain the patient’s Hgb levels and avoid hospital admissions for acute bleeding (Figure 1). As can be seen from the algorithm, all patients undergo upper endoscopy with colonoscopy. If a lesion was not seen, deep enteroscopy was performed for acute bleeding, and video capsule endoscopy was performed for non-acute or chronic bleeding. All patients were placed on iron, NSAIDs and anticoagulants were held if possible, given that many patients were on dual anti-platelet therapy or anticoagulants due to cardiac disease. Lanreotide was given monthly for up to twelve doses in the digestive disease clinic. If patients were not responding to medical management or actively rebled, a repeat capsule and/or deep enteroscopy was performed, depending on the acuteness of the bleeding. 

Since most bleeding lesions appear to be within reach of the push enteroscopy, doing deep enteroscopy would seem to be a prudent first step in such situations. In our algorithm, there is no significant difference between performing deep enteroscopy with a balloon ($1868) or push enteroscopy with an Endocuff ($1751). Capsule endoscopy ($810) costs depend on how many times it is repeated. The major costs for management will come from Lanreotide therapy which can cost from $44,550 to $89,100 depending on whether the patient receives 6–12 doses. We feel push enteroscopy with an Endocuff is superior to deep enteroscopy with a balloon with minor cost savings ($117) depending on whether specialty centers would charge more. Also, push enteroscopy with an Endocuff can be easily performed by most trained gastroenterologists. Lanreotide is a cost breaker with unsure benefits. In our institution, we spent from $1,559,250 to $3,118,500 for our 35 patients that use this drug.

Jackson and Gerson [47] have previously reported on treatments with somatostatin analogs, and the pooled odds ratio was 14.5 (95% CI: 5.9–36) for cessation of bleeding, while the use of hormonal therapy was not effective for bleeding cessation. Garcia–Campean D et al. [41] reported in three studies that using intramuscular Lanreotide at a dose of 10 mg/month to 20 mg/month was effective. The 10 mg/month dose reduced transfusions and hospitalizations, but the 20 mg dose significantly reduced bleeding. However, the numbers were small (13, 11, and 15 patients), with follow-up ranging from 14 to 33 months and with all studies being cohort studies. The beneficial result was a 75–90% reduction in transfusions and iron infusions. A meta-analysis and systemic review of 24 studies involving 831 patients with GIADs showed that octreotide and Lanreotide treatment was more effective than endoscopic therapy for preventing the recurrence of bleeding in both the short and long term [47], but that has not been the experience at our institution.

The endoscopic characteristics of GIADs on capsule endoscopy or deep enteroscopy with regards to bleeding causality and recurrence probability are best described by Garcia-Compean et al. [41]. The type 1, punctuated or patchy lesions with non-pulsatile active bleeding are certain for causality and have a high rebleeding rate without hemostatic treatment. Type 2, non-actively bleeding lesions, stigmata of hemorrhage (ulcer, adherent clot, digested blood debris), have high causality and are highly likely to rebleed. Type 3, bright red spots, have moderate causality with a moderate rebleeding rate. The type 4 lesions (pale red spots) have a low or no causality with a very low re-bleeding rate. As previously described, type 1 and 3 lesions are most frequently found at our institution. Observing the lesion actively bleeding on deep enteroscopy without an Endocuff is infrequent at our institution. These lesions are friable and bleed when touched with a probe (a good technique to use when evaluating lesions).

During the clinic visits for GIADs, we found many of the patients were suffering from medical co-morbidities. The question of how comorbidities influence the incidence or progression of GIADs must be more clearly defined. The presence of comorbidities influences what diagnostic and treatment modalities can be applied. We performed ANOVA analysis on the initial Hgb levels of different co-morbidity indices as co-variance. As expected, we found that the Hgb levels were different between the groups, but the interaction between groups and the co-morbidity index (CMDS) was only marginally significant (*p* = 0.049). These findings suggest that the morbidity Index did not significantly influence the GIADs (Table 3). This finding will require more studies with higher numbers of patients.

Our 12 years of experience with GIADs had a learning curve in management. What we learned is that GIADs are very common in elderly patients with cardiac, pulmonary, renal and liver chronic diseases. Patients typically present with a “herald bleed” and then remain quiescent for up to 2 years. Given this natural history, it is difficult to prove that any therapy is really working, with the exception of those that are actively bleeding. It makes sense to treat those who present with melena with BICAP, clipping, etc., but those with just iron deficiency anemia might only need replacement iron therapy until they are actively bleeding or fail to respond to iron replacement therapy. Somatostatin analogs like Lanreotide are expensive and unpredictable in their effects. In our experience, Lanreotide had no effect. Management of elderly patients with significant comorbidities in our outpatient clinics is conservative unless anemia is progressing, at which time we re-evaluate for endoscopic therapy.

There is no strategy to regress GIADs. There has been no study to show regression of lesions and, when treated with cautery, whether they soon return. An interesting observation from our study is the absence of African American (AA) patients. In our Endocuff GIADs study [3], two AA patients were found. It is unclear whether this is a significant observation, but what is known is that AA patients have a different Vitamin D receptor that binds vitamin D more avidly. At our facility, vitamin D deficiency is very common and exceeds 90%. It might be interesting to observe the frequency of Vitamin D deficiency in our patient population and whether replacement might have an effect on bleeding.

The use of single or double balloon enteroscopy compared to Endocuff enteroscopy for the treatment of GIADs would be interesting, but literature on those who use balloon enteroscopy and the management of GIADs is lacking. Our endoscopists are experienced in both modalities but favor the Endocuff method.

In conclusion, we feel that most patients with symptomatic bleeding small bowel (SB) GIADs can be managed with deep enteroscopy with an Endocuff, with subsequent placement on iron without the use of Lanreotide. We rarely require deep enteroscopy with a balloon. Rebleeding is usually managed by repeating VCE or deep enteroscopy with an Endocuff. We favor performing deep enteroscopy with an Endocuff, which effectively manages our patients. It is noteworthy that the lacey lesions described in the colon and distal small bowel are rarely seen at our institution as the cause of bleeding. This suggests that the need for DAE with a balloon is rarely necessary and should be considered only if DAE with an Endocuff is unsuccessful, if proximal lesions have been treated, significant distal lesions are found, and the patient continues to bleed before sending the patient to radiology or surgery. This would allow the majority of endoscopic practitioners to handle bleeding events from SB GIADs, and if bleeding cannot be controlled, to consider DAE with a balloon. The reduction in cost and greater treatment efficiency is considerable. 

One of the limitations of this study is that the data was generated almost exclusively from the male Veterans who form the majority of our study population. We also have no African Americans in our study. Our future studies will focus on assessing the incidence of GIADs in women and African American Veterans. We will also determine whether Iron treatment alone significantly reduces the recurrence of GIADs in both men and women Veterans. 

## 5. Conclusions

The management of GIADs is complex because of patients’ age and comorbidities. Those patients actively bleeding require more urgent endoscopic therapy, but most individuals can be managed as outpatients. The use of SST or Lanreotide had no significant treatment effect, and it is expensive. The most effective management is to scope and treat active bleeders and manage stable patients receiving iron replacement. Importantly, our data suggest that SST/Lanreotide is not effective in the management of GIADs and not using this treatment would reduce treatment costs. Importantly, the majority of patients can be managed effectively by every practicing gastroenterologist who has standard endoscopy skills.

## Figures and Tables

**Table 1:** Demographic and morbidity index of the study population.

Groups	Ablations	Ablations_SST	Iron	Iron_SST	SST	Observed
Age (Mean ± SEM)	69 ± 1.4	69.7 ± 2.1	68.9 ± 0.85	64.96 ± 1.75	63.7 ± 2.1	63.16 ± 1.60
Ethnicities						
White (A)	48 (A)	18 (A)	106 (A)	19 (A)	6 (A)	49 (A)
Hispanic (B)	11 (B)	05 (B)	20 (2)	05 (B)	1 (B)	08 (B)
Decline (C)	4 (C)	04 (C)	2 (C)	01 (C)	1 (C)	04 (C)
Charlson Morbidity Index (Mean ± SE)	6.49 ± 0.44	7.15 ± 0.61	6.34 ± 0.26	6.52 ± 0.74	6.0 ± 0.69	4.59 ± 0.38
Number of patients	63	27	128	25	8	61

**Table 2:** Hemoglobin level recovery by different therapies at different time points post-treatment.

	Duration	Hemoglobin Levels (Mean ± SEM)					
		Different Therapies					
		Ablations	Ablations_SST	Iron	Iron_SST	SST	Observed
	Initial	11.013 ± 0.33	9.13 ± 0.40	10.59 ± 0.17	10.03 ± 0.61	11.91 ± 1.0	14.20 ± 0.24
Post-treatment duration	Immediate	11.03 ± 0.33	9.13 ± 0.40	10.59 ± 0.17	10.03 ± 0.61	11.90 ± 1.09	14.20 ± 0.24
	1 month	11.91 ± 0.32	10.10 ± 0.36	11.91 ± 0.24 ^A^	10.95 ± 0.50	11.35 ± 1.18	13.72 ± 0.38
	6 months	12.27 ± 031 *	10.84 ± 0.49 ^A^	12.92 ± 0.21 ^A^	11.40 ± 0.51	12.14 ± 0.34	14.12 ± 0.26
	12 months	12.80 ± 0.32 ^A^	10.85 ± 0.50 *	12.89 ± 0.20 ^A^	12.40 ± 0.42 ^A^	13.37 ± 0.87	14.24 ± 0.24

^A^*p* < 0.05 vs. initial (prior to treatment, post hoc test by Tukey) * *p* = 0.06 vs. initial (prior to treatment, Post Hoc test by Tukey).

**Table 3:** Morbidity index influences on GIA.

Source	Type III Sum of Squares	Df	Mean Square	F	Significance
Corrected model	167.622 ^a^	9	18.625	3.814	0.000
Intercept	969.788	1	969.788	198.598	0.000
Group	51.576	4	12.894	2.640	0.035
Cormo_Index	6.430	1	6.430	1.317	0.252
Group_Cormo_Index	47.245	4	11.811	2.419	0.049

^a^. R Squared = 0.125 (Adjusted R Squared = 0.092).

## Data Availability

The data will be unavailable due to VA privacy and ethical restrictions.
